# Systemic corticosteroid use and neurodevelopmental outcomes in preterm infants: a cohort study

**DOI:** 10.1007/s12519-025-00932-4

**Published:** 2025-06-27

**Authors:** Adithya Shastry, Danish Ahmad, Alice Richardson, Barbara Bajuk, Traci-Anne Goyen, Pranav R. Jani, Mohamed E. Abdel-Latif, Neonatal Intensive and Special Care Units’ Data Registry (NICUS)

**Affiliations:** 1 Department of Neonatology, The Australian National University Medical School, Centenary Hospital for Women and Children, Canberra Hospital, PO Box 11, Garran, Canberra, ACT Woden ACT 2606 Australia; 2 School of Medicine and Psychology, College of Science and Medicine, Australian National University, Acton, Canberra, ACT Australia; 3 Statistical Support Network, Australian National University, Acton, Canberra, ACT Australia; 4 NSW Pregnancy and Newborn Services Network, Sydney Children’s Hospitals Network, Randwick, NSW Australia; 5 Department of Neonatology, Westmead Hospital, Westmead, NSW Australia; 6 Reproduction and Perinatal Centre, Faculty of Medicine and Health, The University of Sydney, Sydney, NSW Australia; 7 Discipline of Neonatology, School of Medicine and Psychology, College of Health and Science, Australian National University, Acton, Canberra, ACT Australia

**Keywords:** Neurosensory, Outcome, Preterm, Steroid

## Abstract

**Background:**

The risk–benefit balance and safety of postnatal corticosteroid use for chronic lung disease (PNCSCLD) in preterm infants is a controversial matter. Our objective was to determine the trends in the use of PNCSCLD over eleven years and to analyze the neurodevelopmental consequences of PNCSCLD in preterm infants at 18–42 months of corrected age.

**Methods:**

The data for this retrospective population-based cohort study were obtained from ten tertiary neonatal intensive care units across New South Wales and the Australian Capital Territory, Australia. Preterm infants < 29^+0^ weeks’ gestation born between January 1, 2007, and December 31, 2017, who were alive at discharge, without any major congenital anomalies were included and analyzed based on their PNCSCLD status.

**Results:**

Over eleven years, 611 (14.3%) out of 4258 infants received PNCSCLD. Among the 3386 eligible infants, 2636 (77.8%) underwent neurodevelopmental follow-up and were included in the final analysis. The rate of PNCSCLD use increased from 12.4% in 2007 to 19.6% in 2017. Similarly, the rate of moderate to severe functional disability (MSFD) increased from 8.8% in 2007 to 16.1% in 2017. Propensity-adjusted analysis revealed a greater odds ratio (OR) for MSFD in the PNCSCLD group than in the control group [average treatment effect: OR = 1.252, 95% confidence interval (CI) = 1.185–1.322, *P* ≤ 0.001; average treatment effect on the treated group: OR = 1.104, 95% CI = 1.031–1.184, *P* = 0.005].

**Conclusions:**

PNCSCLD use was associated with a greater incidence of MSFD. Clinicians should exercise caution when using PNCSCLD.

**Graphical Abstract:**

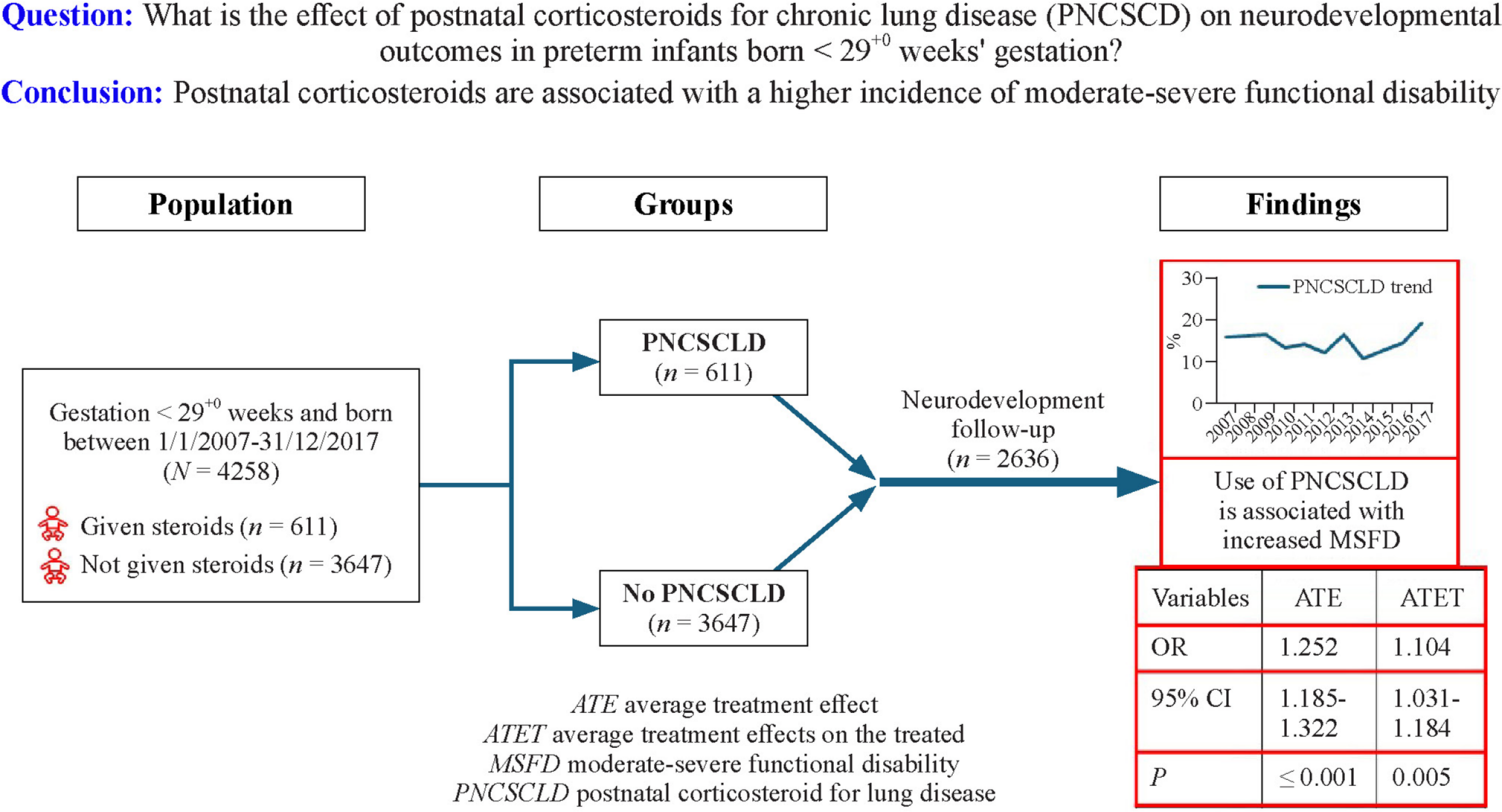

## Introduction

Bronchopulmonary dysplasia (BPD), also known as chronic lung disease (CLD), is a common but disabling complication of prematurity [1]. This condition is typically managed through respiratory support (non-invasive or invasive) [1] and, in some cases, postnatal corticosteroid use for CLD (PNCSCLD) [2]. BPD is associated with long-term morbidities, including developmental [3], language [4], motor [5], and cognitive delays [6, 7], as well as cerebral palsy (CP) [8, 9].

However, the long-term effects of systemic PNCSCLD use on neurodevelopmental outcomes in preterm infants with BPD remain unclear, with conflicting evidence. Ramaswamy et al. reported that PNCSCLD, depending on drug type, dosage and interval, reduced mortality or BPD risk without increasing the risk of neurodevelopmental impairment until 18 to 24 months of age [10]. Furthermore, a Cochrane review suggested that starting PNCSCLD at or after seven days of life could decrease mortality and BPD risk without increasing CP in intubated preterm infants. Still, none of the studies were powered to address adverse long-term neurodevelopmental outcomes [11]. Moreover, prior studies have reported inconsistencies in reporting clear outcomes linked to PNCSCLD use due to high levels of crossover or contamination of steroid use in control groups [11, 12].

Nevertheless, some recent studies have consistently reported an increase in the risk of CP and other complications from the use of PNCSCLD in preterm infants [12]. These conflicting findings have led to variations in BPD management, including the use of PNCSCLD. Clinicians are using PNCSCLD at a lower dosage and for a shorter duration, as well as alternative medications, such as inhaled corticosteroids [13], to minimize long-term complications in preterm infants. A recent meta-analysis highlighted the constant challenge and uncertainty of the risks and harms of PNCSCLD regimens, comparing early versus late treatment with different steroid strengths for overall benefit in the attempt to treat BPD [14]. Independent of the steroid dosage and interval, clearer evidence is needed to evaluate the outcomes associated with the use of PNCSCLD in premature patients.

In two regions in Australia, namely New South Wales (NSW) and the Australian Capital Territory (ACT), over the past decade, the incidence of CLD in infants born at less than 29 weeks' gestation has increased by 15% [15]. Additionally, smaller and less mature infants are being resuscitated and are surviving hospital discharge. The use of the PNCSCLD in this population requires auditing to inform early neurodevelopmental outcomes in clinical practice. This study aimed to review the changing trends in PNCSCLD in preterm infants with BPD and their associations with long-term neurodevelopmental outcomes, including the risk of CP and other complications, across ten neonatal intensive care units (NICUs) in NSW and ACT in Australia. We hypothesize that preterm infants who undergo PNCSCLD have worse outcomes in the neurodevelopmental domain than those who do not.

## Methods

This retrospective population-based cohort study included infants born at less than 29 weeks’ gestational age (23^+0^ to 28^+6^ weeks) between January 1, 2007, and December 31, 2017, who were admitted to NICUs in NSW and the ACT. The data of infants born alive and subsequently admitted to any of the 10 NICUs in NSW and ACT were collected prospectively, verified for standardized definitions by the NICU Audit Officers, and stored in the NICU database. This study extracted hospitalization and follow-up data from the NICUS database.

The inclusion criteria for the study were as follows: (1) gestational age < 29 weeks; (2) no major congenital anomalies that were incompatible with life; (3) alive at discharge at home from the NICU; and (4) followed-up between 18 and 42 months of corrected age (completed between January 22, 2009, and May 7, 2021). We then categorized the data into two groups based on the treatment with postnatal corticosteroids for CLD (PNCSCLD-exposed and control groups).

This study used an administrative database; therefore, informed consent from individual patients was waived by the ACT Health Human Research Ethics Committee (ETHLR.10/344).

### Definitions

CLD was defined as the requirement for respiratory support or supplemental oxygen at 36 weeks of postmenstrual age [16]. Systemic PNCSCLD is given for high-risk babies < 29 weeks’ gestation at risk of CLD at the discretion of the attending neonatologist and treatment in line with standard NICU policies in the ACT and NSW. Inhaled corticosteroids are not considered systemic corticosteroids [17].

### Follow-up neurodevelopmental assessment and tools

All infants who were alive at discharge were assessed at 18–42 months of corrected age. Children were assessed by the developmental assessment team at each of the ten tertiary hospitals. All infants underwent a neurological examination by a trained pediatrician or doctor and a developmental assessment by certified examiners using the Bayley Scales of Infant and Toddler Development, 3rd edition (BSID-III) [18]. Growth measurements (weight, height, and head circumference) and neurosensory information (hearing and vision assessment by an audiologist and optometrist or ophthalmologist, respectively) were also collected at follow-up. These follow-up data are routinely collected at the NSW and ACT for the NICU-discharged preterm neonate database and were accessed for this study.

CP was classified using the Gross Motor Function Classification System (GMFCS) if there was ongoing and nonprogressive motor impairment in a child with abnormal muscle tone, decreased motor control, or any neurological signs [19].

### Outcome measures

The primary outcome of this study was moderate-to-severe functional disability (MSFD) at 18–42 months of age, corrected for prematurity (corrected for gestational age). Functional disability (FD) was classified as follows [19–21]: (1) no FD was defined as BSID-III cognitive, language, and motor composite score above − 1 standard deviation (SD) to below + 3 SD from the mean; (2) mild FD was defined as BSID-III cognitive, language, or motor composite score between − 1 SD and − 2 SD below the mean or mild CP (able to walk without aids, GMFCS level 1) [22]; (3) moderate FD was defined as BSID-III cognitive, language, or motor composite score between − 2 SD and − 3 SD below the mean or moderate CP (able to walk with the assistance of aids, GMFCS levels 2 or 3) or sensorineural or conductive deafness (requiring amplification with bilateral hearing aids or unilateral/bilateral cochlear implants); and (4) severe FD was defined as BSID-III cognitive, language, or motor composite score more than − 3 SD below the mean or bilateral blindness (visual acuity of < 6/60 in the better eye) or severe CP (unable to walk with the assistance of aids, GMFCS levels 4 or 5).

### Statistical analysis

Statistical analyses were performed via Release 28.0.1.0, IBM SPSS Statistics, Chicago, IL, USA, 2021. Propensity score analysis was performed via STATA, version 17, Stata Corp LLC, USA, 2021. Summary statistics are expressed as numbers and percentages (%), medians and interquartile ranges, odds ratios (ORs) with 95% confidence intervals (CIs) and *P* values. The population characteristics of the PNCSCLD and control groups, represented by categorical or continuous data, were compared via the *χ*^2^ test or the Mann‒Whitney *U* test, as appropriate.

Propensity score matching (PSM) was conducted to estimate the adjusted effect of treatment (PNCSCLD) on outcomes by creating matched populations of treated and control patients adjusted for confounders across the antenatal, perinatal and neonatal periods. Propensity adjustments were made to enhance the identification of confounding factors and probable associations in estimating the impact of PNCSCLD on neurodevelopmental outcomes by accounting for covariates selected according to PSM guidelines [23] and the study's aims. The propensity model was generated by first identifying relevant covariates based on clinical relevance and previous literature. These covariates were then included in a logistic regression model to estimate the propensity scores for each individual. The psmatch2 and t-effects commands in Stata 17 were used to perform PSM, estimating the average treatment effect (ATE) and the average treatment effect on the treated (ATET) (StataCorp, 2021). A nearest-neighbour matching method with a 1:1 ratio was used, applying a caliper of 0.2 of the SD of the logit of the propensity score. A 0.2 SD calliper width balances variance and bias, thereby minimizing the mean square error of the estimated treatment effect (Austin, 2011). This ensures that the matched samples are similar, improving the accuracy of the treatment effect estimates. Both ATE and ATET are reported using odds ratios.

The final PSM models accounted for key confounding factors in the cohort by matching treated and control patients with similar propensity scores, thus balancing the distribution of covariates between the groups. This approach helps to control for confounding variables and isolate the effect of PNCSCLD on neurodevelopmental outcomes. While a comprehensive list of confounders was included, "center" was not included in the final model because preliminary analyses indicated that it did not significantly influence the outcomes after adjusting for other covariates. Additionally, including the center as a covariate could have introduced multicollinearity issues, given the already comprehensive list of confounding factors considered. The final PSM models included the following covariates: (1) maternal factors: maternal age, maternal ethnicity, assisted conception, multiple pregnancies, hypertensive disease of pregnancy, gestational diabetes, antenatal corticosteroid exposure, chorioamnionitis and mode of birth; (2) perinatal factors: the place of birth, Apgar score < 7 at 5 minutes, sex, gestational age, birth weight and head circumference < 10th percentile and surfactant; and (3) intermediate neonatal comorbidities, including medically or surgically treated patent ductus arteriosus, grade 3 or 4 intraventricular hemorrhage, grade 3 or 4 retinopathy of prematurity (ROP), medically or surgically diagnosed necrotizing enterocolitis (NEC), and CLD, as these may act, directly or indirectly, as intermediate comorbidities through which the effect of PCNSCLD is mediated in addition to being associated with the outcome.

All analyses were prespecified. All *P* values were two-sided, with *P* < 0.05 considered significant. No adjustment to the significance level was made for multiple comparisons.

## Results

**Fig. 1:**
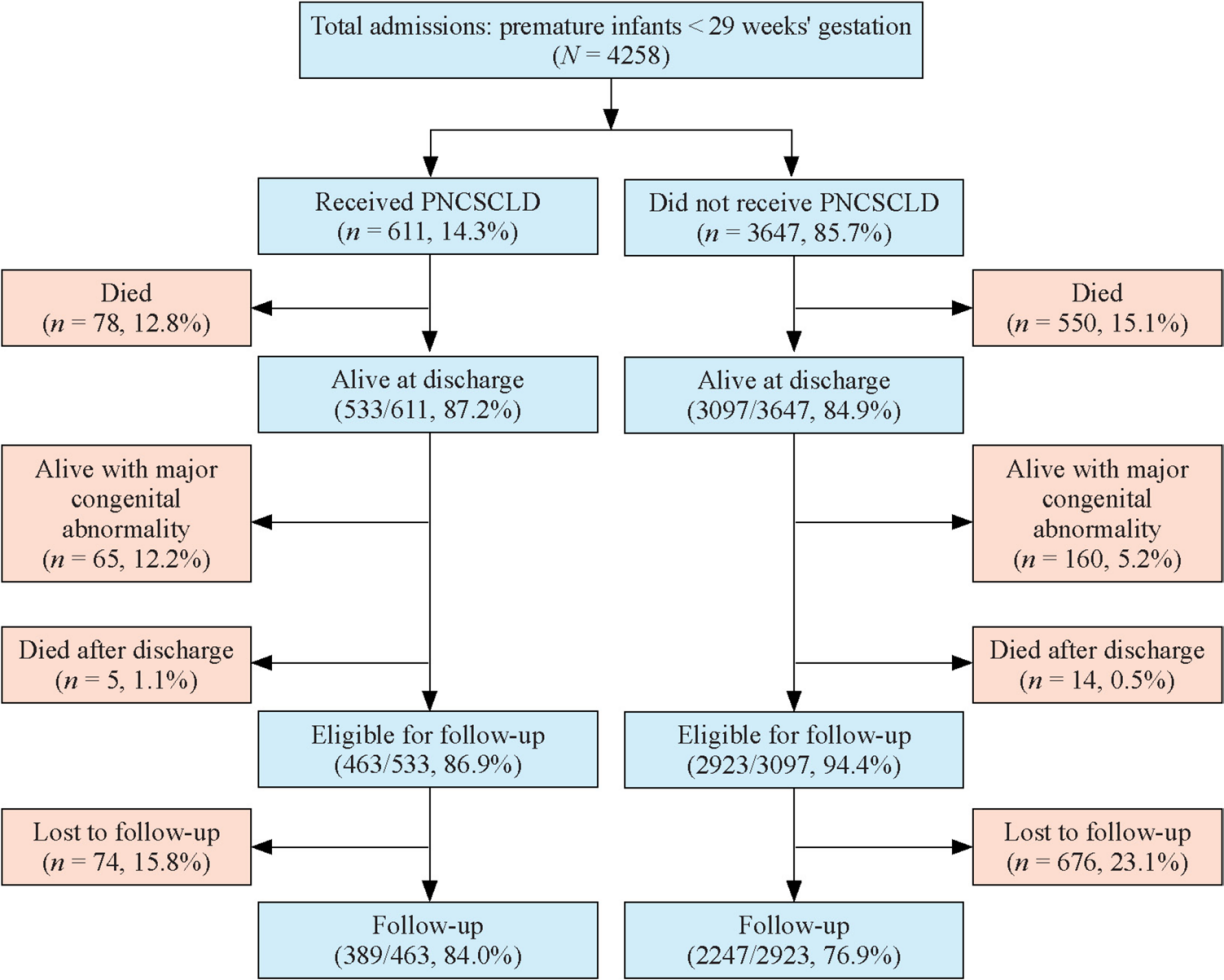
Profile from birth to follow-up of infants treated with and without postnatal corticosteroids for chronic lung disease (PNCSCLD) and admitted to the neonatal intensive care unit between 2007 and 2017

During the study period, a total of 4258 infants were admitted to the NICU. Among these patients, 611 (14.3%) underwent PNCSCLD, and 872 (20.5%) were excluded because of major congenital abnormalities or death before or after discharge. Among the 3386 eligible infants, 2636 (77.8%) completed neurodevelopmental follow-up and were included in the final analysis, while 750 (22.2%) were lost to follow-up (Fig. 1).

### Follow-up and loss to follow-up

Table 1 shows the antenatal and perinatal characteristics of the infants who were followed up and those lost to follow-up. Maternal age was significantly greater in the follow-up group than in the lost-to-follow-up group (31 years vs. 28 years, *P* < 0.001). Assisted conception was less common among infants lost to follow-up (8.5%) than among those in the follow-up group (16%) [unadjusted OR (uOR) = 0.491, 95% CI = 0.372–0.647; *P* < 0.001]. Antenatal health issues, including hypertensive disease during pregnancy (17.9% vs. 13.5%; uOR = 0.715, 95% CI = 0.567–0.902; *P* = 0.005), gestational diabetes (9.9% vs. 7.1%; uOR = 0.692, 95% CI = 0.509–0.941; *P* = 0.022), chorioamnionitis (35.3% vs. 30%; uOR = 0.785, 95% CI = 0.659–0.935; *P* = 0.008) and major surgery (8.9% vs. 6.4%; uOR = 0.699, 95% CI = 0.507–0.964; *P* = 0.034), were significantly more common in the follow-up group. There were no significant differences between the two groups regarding antepartum hemorrhage, multiple pregnancies, cesarean section during labor, vaginal breech, mechanical ventilation, patent ductus arteriosus, intraventricular hemorrhage, NEC or male sex. However, infants born in a nontertiary center were more likely to be lost to follow-up than those born in a tertiary center (16% vs. 7.2%; uOR = 2.466, 95% CI = 1.930–3.151; *P* < 0.001). Neonatal characteristics, including birth weight (and birth weight < 10th percentile), head circumference (< 10th percentile), systemic infection, CLD and ROP, were significantly greater in infants who were followed up (*P* < 0.001).

**Table 1:** Antenatal and perinatal characteristics and major neonatal morbidities among infants based on follow-up status

Characteristics	Followed-up (*n* = 2636)	Lost to follow-up (*n* = 750)	uOR (95% CI)	*P*
Maternal age (y)	31.0 (27.0–35.0)	28.0 (23.0–33.0)	–	< 0.001
Assisted conception	421 (16.0)	64 (8.5)	0.491 (0.372–0.647)	< 0.001
Multiple pregnancy	703 (26.7)	194 (25.9)	0.959 (0.798–1.154)	0.695
Hypertensive disease of pregnancy	471 (17.9)	101 (13.5)	0.715 (0.567–0.902)	0.005
Gestational diabetes	261.0 (9.9)	53.0 (7.1)	0.692 (0.509–0.941)	0.022
Any antenatal corticosteroid	2466 (93.6)	676 (90.1)	0.630 (0.473–0.838)	0.002
Chorioamnionitis^a^	931 (35.3)	225 (30.0)	0.785 (0.659–0.935)	0.008
Vaginal breech	217 (8.2)	55 (7.3)	0.882 (0.649–1.200)	0.470
Cesarean section in labor	1578 (59.9)	446 (59.5)	0.984 (0.834–1.160)	0.878
Born in nontertiary center	189 (7.2)	120 (16.0)	2.466 (1.930–3.151)	< 0.001
Apgar score < 7 at 5 min	688 (26.1)	161 (21.5)	0.774 (0.637–0.940)	0.011
Male sex	1403 (53.2)	397 (52.9)	0.988 (0.840–1.163)	0.921
Gestational age (wk)	27.0 (26.0–28.0)	27.0 (26.0–28.0)	–	< 0.001
Birth weight (g)	940.0 (786.2–1120.0)	1024.5 (890.0–1186.0)	–	< 0.001
Birth weight < 10th percentile	191 (7.2)	20 (2.7)	0.351 (0.220–0.560)	< 0.001
Head circumference (cm)	24.7 (23.2–26.0)	25.4 (24.0–26.5)	–	< 0.001
Head circumference < 10th percentile	81 (3.1)	12 (1.6)	0.513 (0.278–0.946)	0.040
Surfactant therapy	2266 (86.0)	615 (82.0)	0.744 (0.599–0.923)	0.009
Mechanical ventilation	1928 (73.1)	550 (73.3)	1.010 (0.841–1.213)	0.954
Duration of ventilation (d)	2.8 (0.5–11.0)	1.3 (0.4–6.9)	–	< 0.001
Medically or surgically treated patent ductus arteriosus	1234 (46.8)	329 (43.9)	0.888 (0.754–1.045)	0.166
Major surgery	235 (8.9)	48 (6.4)	0.699 (0.507–0.964)	0.034
Proven systemic infection	846 (32.1)	199 (26.5)	0.764 (0.637–0.916)	0.004
Intraventricular hemorrhage grade 3 or 4	108 (4.1)	22 (2.9)	0.707 (0.444–1.127)	0.175
Necrotizing enterocolitis	163 (6.2)	38 (5.1)	0.810 (0.563–1.164)	0.292
Chronic lung disease	1184 (44.9)	251 (33.5)	0.617 (0.520–0.731)	< 0.001
PNCSCLD	389 (14.8)	74 (9.9)	0.632 (0.486–0.823)	< 0.001
Home oxygen	356 (13.5)	41 (5.5)	0.370 (0.265–0.517)	< 0.001
Retinopathy of prematurity stage 3 or 4	328 (12.4)	56 (7.5)	0.568 (0.422–0.763)	< 0.001
Length of intensive care stay (d)	75.1 (57.0–96.6)	65.0 (48.2–83.8)	–	< 0.001

The data are presented as *n* (%), uOR (95% CI), median (interquartile range), and *P* values. The loss to follow-up group was used as a reference for the odds ratio and 95% CI calculations. *PNCSCLD* postnatal corticosteroid use for chronic lung disease, *uOR* unadjusted odds ratio, *CI* confidence interval. ^**a**^Chorioamnitis, including clinically suspected cases. “–” no data

### Trends in postnatal steroid use and neurodevelopmental outcomes

**Fig. 2:**
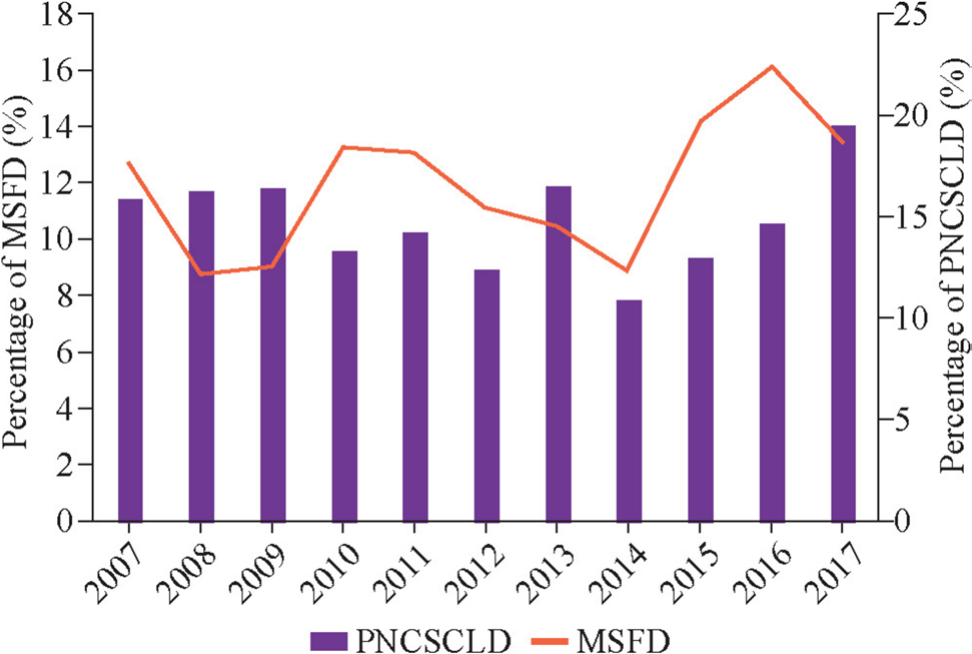
Trends in postnatal corticosteroid use for chronic lung disease (PNCSCLD) and moderate to severe functional disability (MSFD) among infants admitted to the neonatal intensive care unit between 2007 and 2017 across the Australian Capital Territory and New South Wales

The proportion of preterm infants administered PNCSCLD increased from 12.4% in 2007 to 19.6% in 2017 (representing a 7.2% increase). Similarly, the proportion of infants diagnosed with MSFD rose from 8.8% in 2007 to 16.1% in 2017, representing a 7.3% rise (Fig. 2).

### Characteristics of the study groups

Table 2 compares the antenatal and perinatal characteristics of infants who were followed up based on PNCSCLD status. Hypertensive disease during pregnancy, chorioamnionitis, and vaginal breech delivery were significantly more common in the PNCSCLD group (uOR = 1.324, 95% CI = 1.015–1.726, *P* = 0.045; uOR = 1.321, 95% CI = 1.06–1.646, *P* = 0.015; uOR = 1.947, 95% CI = 1.397–2.714, *P* < 0.001), whereas gestational diabetes and cesarean section were more common in the control group. Infants who received PNCSCLD were significantly more likely to have an Apgar score < 7 at 5 minutes of age (38.6% vs. 23.9%; uOR = 1.994, 95% CI = 1.590–2.499; *P* < 0.001), a lower birth weight, birth head circumference, and birth weight < 10th percentile (12.3% vs. 6.4%; uOR = 2.071, 95% CI = 1.465–2.928; *P* < 0.001), required surfactant therapy (97.9% vs. 83.9%; uOR = 9.146, 95% CI = 4.501–18.59; *P* < 0.001), and mechanical ventilation (99.2% vs. 68.6%; uOR = 58.83, 95% CI = 18.83–183.8; *P* < 0.001) for more extended periods (18.5 vs. 1.2 days; *P* < 0.001). Similarly, infants who received PNCSCLDs had significantly greater rates of systemic infection (57.6% vs. 27.7%; uOR = 3.547, 95% CI = 2.843–4.425; *P* < 0.001), PDA requiring medical or surgical treatment (76.6% vs. 41.7%; uOR = 4.587, 95% CI = 3.575–5.885; *P* < 0.001), NEC (14.1% vs. 4.8%; OR = 3.261, 95% CI 2.311–4.603; *P* < 0.001), CLD (88.2% vs. 37.4%; uOR = 12.47, 95% CI = 9.058–17.16; *P* < 0.001) requiring home oxygen (36.8% vs. 9.5%, uOR = 5.551, 95% CI = 4.324–7.126; *P* < 0.001), ROP (37.5% vs. 8.1%; uOR = 6.817, 95% CI = 5.282–8.798; *P* < 0.001), and a prolonged NICU stay (115.5 vs. 70.0, *P* < 0.001).

**Table 2:** Antenatal and perinatal characteristics and major neonatal morbidities among infants born between 2007 and 2017 and assessed at 18 and 42 months of age, corrected for prematurity

Characteristics	Received PNCSCLD (*n* = 389)	Did not receive PNCSCLD (*n* = 2247)	uOR (95% CI)	*P*
Maternal age (y)	31.0 (27.0–35.0)	31.0 (27.0–35.0)	–	0.849
Assisted conception	60 (15.4)	361 (16.1)	0.953 (0.708–1.283)	0.807
Multiple pregnancy	94 (24.4)	609 (27.1)	0.857 (0.667–1.101)	0.251
Hypertensive disease of pregnancy	84 (21.6)	387 (17.2)	1.324 (1.015–1.726)	0.045
Gestational diabetes	24 (6.2)	237 (10.5)	0.558 (0.361–0.861)	0.010
Any antenatal corticosteroid	364 (93.6)	2102 (93.5)	1.004 (0.648–1.558)	0.927
Chorioamnionitis^a^	159 (40.9)	772 (34.4)	1.321 (1.060–1.646)	0.015
Vaginal breech	52 (13.4)	165 (7.3)	1.947 (1.397–2.714)	< 0.001
Cesarean section in labor	210 (54.0)	1368 (60.9)	0.754 (0.607–0.936)	0.012
Born in nontertiary center	23 (5.9)	166 (7.4)	0.788 (0.502–1.235)	0.350
Apgar score < 7 at 5 min	150 (38.6)	538 (23.9)	1.994 (1.590–2.499)	< 0.001
Male sex	237 (60.9)	1166 (51.9)	1.446 (1.160–1.801)	0.001
Gestational age (wk)	25.0 (24.0–26.0)	27.0 (26.0–28.0)	–	< 0.001
Birth weight (g)	743.0 (630.0–890.5)	980.0 (827.0–1150.0)	–	< 0.001
Birth weight < 10th percentile	48 (12.3)	143 (6.4)	2.071 (1.465–2.928)	< 0.001
Head circumference (cm)	23.0 (22.0–24.0)	25.0 (23.6–26.0)	–	< 0.001
Head circumference < 10th percentile	16 (4.1)	65 (2.9)	1.440 (0.824–2.516)	0.260
Surfactant therapy	381 (97.9)	1885 (83.9)	9.146 (4.501–18.59)	< 0.001
Mechanical ventilation	386 (99.2)	1542 (68.6)	58.83 (18.83–183.8)	< 0.001
Duration of ventilation (h)	18.5 (10.1–29.7)	1.2 (0.4–5.3)	–	< 0.001
Medically or surgically treated patent ductus arteriosus	298 (76.6)	936 (41.7)	4.587 (3.575–5.885)	< 0.001
Proven systemic infection	224 (57.6)	622 (27.7)	3.547 (2.843–4.425)	< 0.001
Intraventricular hemorrhage grade 3 or 4	15 (3.9)	93 (4.1)	0.929 (0.533–1.620)	0.903
Necrotizing enterocolitis	55 (14.1)	108 (4.8)	3.261 (2.311–4.603)	< 0.001
Chronic lung disease	343 (88.2)	841 (37.4)	12.47 (9.058–17.16)	< 0.001
Home oxygen	143 (36.8)	213 (9.5)	5.551 (4.324–7.126)	< 0.001
Retinopathy of prematurity stage 3 or 4	146 (37.5)	182 (8.1)	6.817 (5.282–8.798)	< 0.001
Length of intensive care stay, day	115.5 (97.7–137.1)	70.0 (54.1–87.4)	–	< 0.001

The data are presented as *n* (%), uOR (95% CI), median (interquartile range), and *P* values. The no PNCSCLD group was used as a reference for the odds ratio and 95% CI calculations. *PNCSCLD* postnatal corticosteroid use for chronic lung disease, *uOR* unadjusted odds ratio, *CI* confidence interval. ^**a**^Chorioamnionitis, including clinically suspected cases. “–” no data

### Neurodevelopmental outcomes and postnatal steroid use

#### Unadjusted analysis

Infants who received PNCSCLD (*n* = 389) were more likely to have abnormal scores in the cognitive, language, and motor domains, including the moderate-severe range (Table 3). These included MSFD (26% vs. 9.6%; uOR = 3.311, 95% CI = 2.536–4.323; *P* < 0.001), cognitive delay (7.6% vs. 2.2%; uOR = 3.706, 95% CI = 2.285–6.010; *P* < 0.001), language delay (18.3% vs. 6.1%; uOR = 3.440, 95% CI = 2.498–4.737; *P* < 0.001), motor delay (12.7% vs. 3.8%; uOR = 3.805, 95% CI = 2.601–5.567; *P* < 0.001), bilateral blindness (1.8% vs. 0.3%; uOR = 6.844, 95% CI = 2.288–20.470; *P* < 0.001), and bilateral deafness (3.6% vs. 0.9%; uOR = 4.157, 95% CI = 2.082–8.302; *P* < 0.001). The only exception was moderate to severe CP (3.1% vs 1.7%; uOR = 1.802, 95% CI = 0.935–3.473; *P* = 0.113).

**Table 3:** Neurodevelopmental and anthropometric outcomes of infants born between 2007 and 2017 and assessed at 18–42 months of age, corrected for prematurity

Characteristics		Received PNCSCLD (n = 389)	Did not receive PNCSCLD (n = 2247)	uOR (95% CI)	*P*
Postnatal age at assessment (mon)		26.46 (24.18–34.53)	26.16 (24.33–34.07)	–	0.942
Functional disability					
None/minimal		168 (43.2)	1490/2245 (66.4)	0.385 (0.310–0.479)	< 0.001
Mild		120 (30.8)	540/2245 (24.1)	1.409 (1.113–1.783)	0.005
Moderate		70 (18.0)	157/2245 (7.0)	2.918 (2.151–3.960)	< 0.001
Severe		31 (8.0)	58/2245 (2.6)	3.265 (2.082–5.121)	< 0.001
Moderate to severe		101 (26.0)	215/2245 (9.6)	3.311 (2.536–4.323)	< 0.001
Cognitive composite score					
Normal or better (0 SD to − 1 SD)		281/369 (76.2)	1894/2122 (89.3)	0.384 (0.292–0.507)	< 0.001
Mild (− 1 SD to − 2 SD)		61/369 (16.5)	182/2122 (8.6)	2.111 (1.542–2.890)	< 0.001
Moderate (− 2 SD to − 3 SD)		28/369 (7.6)	46/2122 (2.2)	3.706 (2.285–6.010)	< 0.001
Severe (> − 3 SD)		0	0	–	–
Moderate to severe		28/369 (7.6)	46/2122 (2.2)	3.706 (2.285–6.010)	< 0.001
Language composite score					
Normal		215/361 (59.6)	1595/2064 (77.3)	0.433 (0.343–0.547)	< 0.001
Mild		80/361 (22.2)	343/2064 (16.6)	1.428 (1.086–1.879)	0.013
Moderate		45/361 (12.5)	95/2064 (4.6)	2.952 (2.031–4.290)	< 0.001
Severe		21/361 (5.8)	31/2064 (1.5)	4.051 (2.301–7.132)	< 0.001
Moderate to severe		66/361 (18.3)	126/2064 (6.1)	3.440 (2.498–4.737)	< 0.001
Motor composite score					
Normal		239/368 (64.9)	1744/2105 (82.9)	0.384 (0.301–0.489)	< 0.001
Mild		82/368 (22.3)	283/2105 (13.4)	1.846 (1.401–2.432)	< 0.001
Moderate		34/368 (9.2)	60/2105 (2.9)	3.470 (2.243–5.367)	< 0.001
Severe		13/368 (3.5)	18/2105 (0.9)	4.246 (2.062–8.742)	< 0.001
Moderate to severe		47/368 (12.7)	78/2105 (3.8)	3.805 (2.601–5.567)	< 0.001
Cerebral palsy					
Mild		28/389 (7.2)	81/2247 (3.6)	2.074 (1.331–3.233)	0.002
Moderate		4/389 (1.0)	13/2247 (0.58)	1.785 (0.579–5.504)	0.497
Severe		8/389 (2.1)	26/2247 (1.2)	1.794 (0.806–3.991)	0.228
Moderate to severe		12/389 (3.1)	39/2247 (1.7)	1.802 (0.935–3.473)	0.113
Any		40/389 (10.3)	120/2247 (5.3)	2.032 (1.396–2.957)	< 0.001
Bilateral blindness		7 (1.8)	6 (0.3)	6.844 (2.288–20.470)	< 0.001
Bilateral deafness		14 (3.6)	20 (0.9)	4.157 (2.082–8.302)	< 0.001
Weight (kg)		12.1 (10.7–13.5)	12.6 (11.4–14.0)	–	< 0.001
Height (cm)		87.5 (84.0–92.1)	89.0 (85.0–93.0)	–	< 0.001
Head circumference (cm)		47.5 (46.0–48.7)	48.5 (47.0–49.6)	–	< 0.001

The data are presented as *n* (%), uOR (95% CI), median (interquartile range), and *P* values. The no PNCSCLD group was used as a reference for the odds ratio and 95% CI calculations. The denominator is the number of infants examined. *PNCSCLD* postnatal corticosteroid use for chronic lung disease, *SD* standard deviation, *uOR* unadjusted odds ratio, *CI* confidence interval. “–” no data

### Propensity-adjusted average treatment effect and average treatment effect on the treated

Table 4 shows the propensity-adjusted differences in neurodevelopmental outcomes at follow-up after correction for key maternal and perinatal factors and neonatal comorbidities. Both the ATE and ATET of the PNCSCLD group were greater than those of the control group [adjusted OR (aOR) = 1.252, 95% CI = 1.185–1.322, *P* ≤ 0.001; aOR = 1.104, 95% CI = 1.031–1.184, *P* = 0.005, respectively]. The odds for moderate to severe CP increased for infants with birth weights < 1000 g (ATE: OR = 1.050, 95% CI = 1.015–1.085, *P* = 0.004; ATET: OR = 1.711, 95% CI = 1.019–1.091, *P* = 0.002, respectively) (Table 4).

**Table 4:** Unadjusted and propensity-adjusted neurodevelopmental outcomes of infants who received PNCSCLD between 2007 and 2017 and who were assessed at 18–42 months of age, corrected for prematurity

Characteristics	Unadjusted OR (95% CI); *P*	Adjusted OR (95% CI); *P*^a^	
		ATE	ATET
Moderate to severe functional disability	3.311 (2.536–4.323); < 0.001	1.252 (1.185–1.322); < 0.001	1.104 (1.031–1.184); 0.005
Moderate to severe cognitive delay	3.706 (2.285–6.010); < 0.001	1.140 (0.973–1.336); 0.106	1.015 (1.014–1.091); 0.008
Moderate to severe language delay	3.440 (2.498–4.737); < 0.001	1.273 (1.191–1.359); < 0.001	1.077 (1.014–1.014); 0.017
Moderate to severe motor delay	3.805 (2.601–5.567); < 0.001	1.225 (1.133–1.324); < 0.001	1.051 (1.004–1.101); 0.032
Moderate to severe cerebral palsy	1.802 (0.935–3.473); 0.113	1.134 (0.977–1.318); 0.098	1.019 (0.991–1.047); 0.196
Any cerebral palsy	2.013 (1.384–2.929); < 0.001	1.139 (0.970–1.338); 0.113	1.037 (0.996–1.079); 0.082
Cerebral palsy in < 1000 g BW	2.191 (1.416–3.389); < 0.001	1.050 (1.015–1.085); 0.004	1.711 (1.019–1.091); 0.002
Bilateral blindness	6.844 (2.288–20.470); < 0.001	0.872 (0.743–1.023); 0.094	0.995 (0.975–1.015); 0.604
Bilateral deafness	4.157 (2.082–8.302); < 0.001	1.014 (1.000–1.029); 0.055	1.032 (0.999–1.062); 0.058

The data were analyzed for 389 matched infants receiving PNCSCLD with controls (778 in total) and are presented as OR (95% CI) and *P* values. The “no PNCSCLD” group was used as a reference for the odds ratio and 95% CI calculations. *ATE* average treatment effect, *ATET* average treatment effects on the treated, *PNCSCLD* postnatal corticosteroid use for chronic lung disease, *BW* birth weight, *OR* odds ratio, *CI* confidence interval. ^a^Adjusted for (1) maternal and perinatal factors: maternal age, maternal ethnicity, assisted conception, multiple pregnancies, hypertensive disease of pregnancy, gestational diabetes, antenatal corticosteroid use, chorioamnionitis, mode of birth, place of birth, Apgar score < 7 at 5 minutes, sex, gestational age, birth weight and head circumference < 10th centiles and surfactant; and (2) intermediate comorbidities: medically or surgically treated patent ductus arteriosus, proven systemic infection, grade 3 or 4 intraventricular hemorrhage, radiologically or surgically diagnosed necrotizing enterocolitis, grade 3 or 4 retinopathy of prematurity, using propensity score matching analysis

### Timing and doses of postnatal steroids

Data on the timing of PNCSCLD were available for 362 out of 389 (93.0%) infants who received the treatment (Table 5). Specifically, 14 (3.9%) infants received early steroids, which were administered within the first 7 days of life, while the remaining infants received delayed steroids, administered after the first 7 days. Due to the small sample size, a detailed analysis could not be performed (Table 5). Additionally, information regarding the dosages of PNCSCLD was not available.

**Table 5:** Comparisons of neurodevelopmental outcomes in infants given early or delayed corticosteroids or no corticosteroids for chronic lung disease

Characteristics	Not given PNCSLD (*n* = 2247)	Early (≤ 7 d of life) (*n* = 14)	Delayed (> 7 d of life) (*n* = 348)
Moderate to severe functional disability	215/2245 (9.6)	1/14 (7.1)	94/348 (27.0)
Moderate to severe cognitive delay	46/2122 (2.2)	1/14 (7.1)	26/334 (7.8)
Moderate to severe language delay	126/2064 (6.1)	1/14 (7.1)	64/326 (19.6)
Moderate to severe motor delay	78/2105 (3.8)	–	9/35 (25.7)
Moderate to severe cerebral palsy	120/2247 (5.3)	–	9/35 (25.7)
Cerebral palsy (any)	279/2247 (12.4)	1/35 (2.9)	35/348 (10.1)
Bilateral blindness	6 (0.3)	0/14	5/348 (1.4)
Bilateral deafness	20 (0.9)	0/14	13/348 (3.7)

*PNCSCLD* postnatal corticosteroid use for chronic lung disease. “–” no data

## Discussion

The clinical use of PNCSCLD has been controversial for many years due to associated complications [14]. The present study investigated the associations between PNCSCLD use and neurodevelopmental outcomes in a cohort of preterm infants born between 2007 and 2017 at the NSW and the ACT in Australia.

The rate of PNCSCLD increased by 7.2% from 2007–2017, broadly reflecting the increased survival of infants at the border of viability in NICUs of ACT and NSW. Similarly, the proportion of infants diagnosed with MSFD increased by 7.3% during the same period. However, the rate of PNCSCLD use did not always correlate with the proportion of MSFD at follow-up, possibly indicating the influence of other factors. Our results also demonstrated that infants who received PNCSCLD had significantly poorer neurodevelopmental outcomes, including FD, cognitive delay, language delay, bilateral blindness, and bilateral deafness, after controlling for key maternal, perinatal and neonatal confounders.

The ATE measures the average treatment effect of the PNCLSD on the whole population by comparing neonates who received the PNCLSD to those who did not receive postnatal steroids. On average, the use of postnatal steroids was associated with greater MSFD among neonates in the study population regardless of individual treatment status. The ATET results enable us to capture the average treatment effect of the PNCLSD on outcomes among neonates who received the treatment by comparing it against the counterfactual, i.e., what would have happened if the treatment group had not received it. The ATET compares treated neonates against counterfactuals or matched controls (using the same covariates as ATE) who would otherwise have been eligible to receive steroids. The ATET results revealed that, on average, among neonates who were treated with steroids, there was a greater likelihood of worse outcomes than among those who were not treated with steroids.

These findings suggest that PNCSCLD use may adversely impact neurodevelopment in this vulnerable population. The rates of adverse effects of PNCSCLD on neurodevelopmental outcomes are consistent with those reported in previous studies, including several randomized controlled trials and observational studies in preterm infants [23–25]. Our data suggest that PNCSCLD may increase the odds of CP in infants with a birth weight of less than 1000 g. Similar findings were reported in a recent Australian study [19]. Taken together, clinicians should use a cautious approach to using PNCSCLD in this population.

In addition to the primary outcome, we compared the antenatal and perinatal characteristics of the infants who were followed up with those of those who were lost to follow-up. Notably, infants born in a nontertiary center were more likely to be lost to follow-up, highlighting the importance of effective communication and facilitating ongoing care between different healthcare facilities and multidisciplinary teams to ensure continuity of care, as indicated by previous research [26].

The comparison of infants based on PNCSCLD status revealed several notable differences in antenatal and perinatal characteristics. The mothers of infants in the PNCSCLD group had greater incidences of hypertensive disease during pregnancy, chorioamnionitis, and vaginal breech. On the other hand, the mothers in the control group had a greater incidence of gestational diabetes and cesarean section, which may reflect different risk factor profiles and perinatal complications.

Regarding neonatal characteristics, infants who received PNCSCLD had lower Apgar scores at 5 minutes, indicating a greater risk of immediate neonatal compromise. They also exhibited a higher rate of intrauterine growth restriction, characterized by lower birth weights and head circumferences, as well as a greater incidence of being small for gestational age. Additionally, infants who received PNCSCLD had greater ventilatory support, including surfactant therapy and mechanical ventilation, reflecting a greater burden of respiratory morbidity. This observation aligns with previous research indicating that PNCSCLD is often administered to infants with significant respiratory distress syndrome or other pulmonary conditions [2].

Furthermore, infants who received postnatal PNCSCLD had increased rates of systemic infection, patent ductus arteriosus requiring treatment, NEC, CLD requiring home oxygen, and ROP. This suggests that infants who received PNCSCLD treatment were more likely to be sicker than those in the control group. The prolonged NICU stay with mechanical ventilation observed in the PNCSCLD group further supports the likelihood that these infants had a more severe clinical course and required more intensive medical interventions for survival. We employed robust statistical modeling and propensity score matching (PSM) analysis to account for baseline differences between the PNCSCLD exposure group and the control group.

The strengths of our study include (1) the use of a uniform geographic cohort reflecting the Australian preterm population; (2) the use of longitudinal, prospectively collected and audited data using standardized definitions for clinical outcomes; and (3) the use of PSM analysis.

It is important to acknowledge several limitations of this study. The dose of the steroids used was difficult to determine in our research, as these data were not collected. The clinical correlation between the dose and timing of PNCSCLD and neurodevelopment at follow-up has been studied elsewhere [14, 27]. Ideally, the timing of postnatal corticosteroid administration should maximize pulmonary benefits without increasing adverse neurodevelopmental risks. Unintended steroid exposure in the control group is another important aspect. We did not have information on the use of other steroids, such as hydrocortisone, for hypotension.

Furthermore, the loss to follow-up of a considerable proportion of infants may introduce selection bias and potentially affect the generalizability of the results. Although efforts have been made to adjust for confounding factors, residual confounding may still exist owing to unmeasured or imperfectly measured confounders. This means that the observed associations may not necessarily imply causation [27], as some confounding factors might not have been fully accounted for. Although we used standardized tools for developmental assessments, not all NICUs in the study adopted the BSID III at the same time. Finally, we were unable to provide details on respiratory support status (invasive versus non-invasive ventilation) when PNCSCLD was used or its impact on neurodevelopment.

In conclusion, this study provides evidence that PNCSCLD use in preterm infants is associated with a greater risk of adverse neurodevelopmental outcomes. This study underscores the importance of carefully considering the benefits and risks of PNCSCLD in neonatal healthcare. Future research should focus on the mechanisms by which PNCSCLD impacts neurodevelopment, its impact on long-term neurodevelopment when used in infants receiving non-invasive ventilation, and alternative methods for treating respiratory disease in preterm infants to minimize long-term neurodevelopmental impairment.

## Data Availability

The data may be obtained from a third party and are not publicly available. The data for this study were extracted from the Neonatal Intensive and Special Care Units' Data Registry (NICUS), an ongoing prospective statewide audit of infants admitted to the 10 NICUs in the NSW and the ACT during the neonatal period. The authors will not be able to share any individual participant data. However, the data may be available upon reasonable request from the NICUS Data Custodian, Agency for Clinical Innovation, NSW, Australia. All data relevant to the study are included in the article or uploaded as supplementary information.
